# Emerging and legacy PFAS and cytokine homeostasis in women of childbearing age

**DOI:** 10.1038/s41598-022-10501-8

**Published:** 2022-04-20

**Authors:** Min Nian, Wei Zhou, Yan Feng, Yan Wang, Qian Chen, Jun Zhang

**Affiliations:** 1 Ministry of Education-Shanghai Key Laboratory of Children’s Environmental Health, School of Public Health, Shanghai Jiao Tong University School of Medicine, Shanghai, 200025 China; 2 Center for Reproductive Medicine, Cheeloo College of Medicine, Shandong University, Jinan, 250012 Shandong China; 3 Key Laboratory of Reproductive Endocrinology of Ministry of Education, Shandong University, Jinan, 250012 Shandong China; 4 Ministry of Education-Shanghai Key Laboratory of Children’s Environmental Health, Xinhua Hospital, Shanghai Jiao-Tong University School of Medicine, No.1665, Kongjiang Road, Shanghai, 200092 China; 5 The Ninth People’s Hospital of Shanghai Jiao Tong University School of Medicine, Shanghai, 200011 China

**Keywords:** Cytokines, Environmental sciences, Risk factors

## Abstract

Per- and polyfluoroalkyl substances (PFAS) are widespread chemicals. Legacy PFAS have been phased out of production in most developed countries and emerging PFAS (short-chain PFAS and polyfluorinated compounds) are used as legacy PFAS alternatives. The effect of legacy and emerging PFAS on cytokine homeostasis in human remains poorly understood. This study aimed to evaluate the associations between legacy and emerging PFAS and cytokine profiles, and identify the main contributors to the disturbance of cytokine homeostasis. We quantified 21 PFAS in 198 Chinese women of childbearing age from 2015 to 2016. 13 cytokines were measured using the Meso Scale Discovery U-PLEX and V-PLEX platforms. The associations between PFAS exposure and cytokine levels were assessed using multiple linear regression (single-exposure), and Bayesian kernel machine regression (BKMR) models (PFAS mixture exposure). In single PFAS models, legacy and alternative PFAS were positively associated with Th1 and Treg cytokines, and negatively associated with Th2 and Th17 cytokines. For instance, each ln-unit increase in 6:2 chlorinated perfluoroalkyl ether sulfonic acid (6:2 Cl-PFESA), perfluorooctanoic acid (PFOA), and perfluorooctane sulfonate (PFOS) was associated with a decrease in IL-10 by − 0.228 (95% CI: − 0.336, − 0.120), − 0.153 (95% CI: − 0.277, − 0.030), and − 0.174 (95% CI: − 0.339, − 0.010), respectively. The BKMR model showed a significantly positive association of PFAS mixture with TGF-β and a negative association with IL-10. Overall, these results indicate that both legacy and emerging PFAS may affect the homeostasis of cytokines.

## Introduction

Per- and polyfluoroalkyl substances (PFAS) are a class of synthetic compounds that have been used in many industrial and consumer applications for over 70 years because of their high thermal and chemical stability^1^. Perfluorooctane sulfonate (PFOS) and perfluorooctanoic acid (PFOA) are two of the most studied long-chain PFAS, and are reported to induce adverse effects on human, including immunotoxicity^2^, carcinogenicity^3^, and reproductive and developmental toxicity^4^. Due to their persistence in the environment and potential health implications in humans, PFOS and PFOA production and applications have been restricted in Europe and North America^5,6^. Consequently, PFAS alternatives with either a short chain (perfluorosulfonic acid with carbon number < 6 and perfluorocarboxylic acid with carbon number < 8^1^) or a slightly modified structure have emerged to meet market demand^7^. These alternatives are increasingly found in environmental and human samples at relatively high concentration due to the unrestricted use. For example, 6:2 chlorinated perfluoroalkyl ether sulfonic acid (6:2 Cl-PFESA) concentrations were as high as 2.13 ng/mL in Shenyang population, China^8^, and have become one of the main PFAS in Chinese local surface waters^9^. And PFAS alternatives showed comparable or even greater toxicities in both epidemiological and toxicological studies^8,10,11^. However, literature on immunotoxicity of PFAS alternatives is still scarce and inconsistent.

The immune system plays a critical role in human reproduction^12^. The balance between pro- and anti-inflammatory cytokines in female immune system is essential for embryo implantation and pregnancy maintenance. T-helper 1 (Th1) and Th2 cells are the major subsets of Th cells with different patterns of cytokine production and different roles in immune responses^13^. In female, the deviation of the immune system from Th2 toward Th1 has been implicated in pregnancy complications, such as recurrent miscarriage^13,14^, preeclampsia^15^ and fetal growth restriction^16^. Th1 cells secrete pro-inflammatory cytokines such as interferon-gamma (IFN-γ), tumor necrosis factor-alpha (TNF-α), interleukin-2 (IL-2), IL-8, and IL-12p70, that can activate macrophages and cell-mediated reactions that play critical roles in resistance to infection by intracellular pathogens and in cytotoxic and rejection reactions. Th2 cells secrete IL-4, IL-6, IL-10, and IL-13, which induce antibody production and are associated with strong humoral immunity^17^. IL-17 and IL-22 are the main Th17 effector cytokines, which are related to protective immunity against extracellular microbes during pregnancy^13,18^. Transforming growth factor-β (TGF-β) is secreted by regulatory T (Treg) cells and has the function of local inflammatory immune responses, potentially detrimental to the fetus^13^. Epidemiological findings regarding the possible relationship of PFAS and immune cytokines are limited and inconsistent. A recent study in 87 middle-aged women found that PFHpS was positively associated with Th1 cytokine (IL-8)^19^. But the C8 Health Project found that PFHxS, PFOA, and PFOS were inversely associated with TNF-α in 200 women aged 40–70 years. Similarly, PFOS and PFNA were negatively associated with IL-8^20^. The inconsistency of previous findings warrants further research. We speculated that PFAS might affect pregnancy by disrupting immune balance^14,19^.

We conducted a cross-sectional study to examine the association between legacy and emerging PFAS exposure and cytokine levels and their balances.

## Results

### Participant characteristics

A total of 198 women were included in our study. Table 1 shows characteristics of the study population. The mean age was 27.7 years old and average years at menarche was 14.2 years old. Almost 21.1% of participants had college education.

**Table1:** Characteristics of participants.

Characteristic	n	Mean ± SD or %
Age (year)	198	27.7 ± 3.5
BMI (kg/m^2^)	198	23.2 ± 3.8
Age at menarche (year)	198	14.2 ± 1.6
**Education**		
Primary school	22	11.1
Middle school	134	67.7
Above middle school	42	21.2

Table 2 summarizes the concentrations of PFAS measured in the plasma of the participants. PFOA (3.94 ng/mL), PFOS (3.37 ng/mL), n-PFOS (2.32 ng/mL) and 6:2 Cl-PFESA (1.40 ng/mL) had the highest concentrations. The correlation coefficients among the PFAS ranged from 0.02 to 0.89 (see Supplementary Fig. S1).

**Table 2:** Distribution of plasma PFAS.

PFAS (ng/mL)	Detection rate (%)	25th	50th	75th	Mean	SD
n-PFHxS	100	0.115	0.155	0.269	0.234	0.265
Br-PFHxS	69.7	0.007	0.012	0.015	0.014	0.018
6 m-PFOS	100.0	0.153	0.238	0.362	0.317	0.278
1 m-PFOS	100.0	0.067	0.104	0.163	0.131	0.101
∑3,4,5 m-PFOS	100.0	0.254	0.401	0.924	0.645	0.566
Br-PFOS	100.0	0.486	0.752	1.605	1.093	0.865
n-PFOS	100.0	1.434	2.321	3.883	3.277	3.061
6:2 Cl-PFESA	100.0	0.741	1.403	2.402	2.226	4.021
8:2 Cl-PFESA	100.0	0.036	0.060	0.078	0.065	0.051
HFPO-DA	95.5	0.007	0.011	0.033	0.020	0.016
PFBS	80.8	0.011	0.022	0.049	0.042	0.056
PFHpA	96.5	0.029	0.051	0.101	0.079	0.105
PFBA	96.0	0.065	0.095	0.121	0.136	0.631
PFHxA	93.9	0.009	0.017	0.041	0.043	0.082
PFOA	100.0	2.406	3.938	7.645	8.566	14.420
PFOS	100.0	2.094	3.371	5.540	4.370	3.711
PFNA	100.0	0.315	0.473	0.739	0.652	0.571
PFDA	100.0	0.218	0.369	0.653	0.514	0.499
PFHxS	100.0	0.126	0.167	0.281	0.249	0.280
PFHpS	100.0	0.058	0.081	0.105	0.088	0.047
PFDoA	100.0	0.065	0.092	0.131	0.269	1.321
PFUdA	100.0	0.206	0.320	0.566	0.427	0.325

Table 3 presents the distribution of 13 cytokines in all the participants’ serum samples. Median concentrations of IFN-γ, TNF-α, IL-12p70, IL-8, IL-6, IL-10, IL-17, IL-22 were 6.12, 1.19, 0.05, 8.21, 0.40, 0.13, 0.80, 0.31 ng/ml, respectively, and TGF-β was 17.4 μg/mL, respectively. The detection rates of IL-1β (33.8%), IL-2 (26.8%), IL-4 (6.1%) and IL-3 (27.3%) were too low to perform further statistical analysis.

**Table 3:** Serum cytokine concentrations in participants (n = 198).

	Detection rate (%)	25th	50th	75th	Mean	SD
**Th1 cytokines**						
IL-1β (ng/mL)	33.8	0.01	0.02	0.05	0.06	0.16
IL-2 (ng/mL)	26.8	0.02	0.06	0.13	0.12	0.24
IL-8 (ng/mL)	100.0	6.1	8.21	10.89	14.69	32.35
IL-12p70 (ng/mL)	41.4	0.02	0.05	0.09	0.08	0.09
TNF-α (ng/mL)	100.0	0.91	1.19	1.54	1.24	0.47
IFN-γ (ng/mL)	100.0	4.21	6.12	10.49	10.23	17.50
**Th2 cytokines**						
IL-4 (ng/mL)	6.1	0.002	0.006	0.009	0.015	0.03
IL-6 (ng/mL)	100.0	0.25	0.40	0.62	0.50	0.37
IL-10 (ng/mL)	100.0	0.08	0.13	0.21	0.25	0.81
IL-13 (ng/mL)	27.3	0.10	0.18	0.46	0.35	0.54
**Th17 cytokines**						
IL-17 (ng/mL)	100.0	0.46	0.80	1.34	1.07	1.23
IL-22 (ng/mL)	94.4	0.14	0.31	0.69	0.97	3.34
**Treg cytokines**						
TGF-β (μg/mL)	100.0	14.35	17.4	21.57	18.11	5.40

### Multiple linear regressions

Tables 4 and S2 present linear regression between single PFAS exposure and cytokines. For one ln-unit increase in 1 m-PFOS was associated with a 0.177 increase in IL-8 level (95% CI: 0.031, 0.324). Similarly, one scaled unit increase in ln-transformed 6 m-PFOS, ∑3,4,5 m-PFOS, n-PFOS and PFOS, a 0.370 (95% CI: 0.091, 0.650), 0.282 (95% CI: 0.034, 0.531), 0.354 (95% CI: 0.029, 0.678), and 0.364 (95% CI: 0.005, 0.723) ln-unit increase in IL-12p70 level, respectively. As for the Th2 cytokines, ln-(n-PFOS), ln-(6:2 Cl-PFESA), ln-PFOA, and ln-PFOS concentrations were significantly associated with a decreased level of IL-10 [n-PFOS: β = − 0.175, (95% CI: − 0.322, − 0.028); 6:2 Cl-PFESA: β = − 0.228, (95% CI: − 0.336, − 0.120); PFOA: β = − 0.153, (95% CI: − 0.277, − 0.030); PFOS: β = − 0.174, (95% CI: − 0.339, − 0.010)] in covariate-adjusted models. In addition, lower serum IL-17 levels were observed for each log-unit increase in 8:2 Cl-PFESA (β =  − 0.285, 95% CI: − 0.527, − 0.042). Conversely, each ln-increase in n-PFHxS [β = 0.082 (95% CI: 0.007, 0.157)] and PFOA [β = 0.06 (95% CI: 0.008, 0.111)] were associated with an increase in TGF-β. The similar findings of the relationship between each PFAS and cytokines are presented in Supplementary Tables S3-S8. For instance, the highest exposure quantile of 6:2 Cl-PFESA had a decreased IL-10 level compared to the lowest quantile [β = − 0.66, 95% CI: (− 0.98, − 0.33), *P* for trend < 0.001).

**Table 4:** Associations between single ln-transformed PFAS and cytokines in multivariable linear regression (n = 198)*.

PFAS	Th1				Th2		Th17		Treg
	IL-8	IL-12p70	TNF-α	IFN-γ	IL-6	IL-10	IL-17	IL-22	TGF-β
n-PFHxS	− 0.053 (− 0.205, 0.100)	0.294 (− 0.072, 0.660)	0.029 (− 0.052, 0.110)	0.144 (− 0.027, 0.315)	0.064 (− 0.072, 0.201)	− 0.173 (− 0.354, 0.007)	0.025 (− 0.179, 0.229)	0.055 (− 0.239, 0.348)	**0.082 (0.007, 0.157)**
Br-PFHxS	− 0.125 (− 0.315, 0.064)	0.317 (− 0.124, 0.759)	− 0.006 (− 0.106, 0.095)	− 0.005 (− 0.219, 0.208)	− 0.083 (− 0.252, 0.086)	− 0.224 (− 0.448, 0.000)	− 0.015 (− 0.270, 0.240)	− 0.052 (− 0.427, 0.323)	0.030 (− 0.065, 0.124)
6 m-PFOS	0.063 (− 0.068, 0.195)	**0.370 (0.091, 0.650)**	− 0.018 (− 0.088, 0.052)	0.025 (− 0.123, 0.173)	− 0.037 (− 0.155, 0.081)	− 0.150 (− 0.305, 0.006)	0.060 (− 0.117, 0.236)	− 0.029 (− 0.277, 0.220)	0.041 (− 0.024, 0.106)
1 m-PFOS	**0.177 (0.031, 0.324)**	− 0.135 (− 0.486, 0.215)	0.017 (− 0.062, 0.096)	0.040 (− 0.128, 0.208)	− 0.008 (− 0.141, 0.125)	− 0.066 (− 0.244, 0.111)	− 0.054 (− 0.253, 0.145)	0.150 (− 0.131, 0.431)	0.013 (− 0.061, 0.087)
∑3,4,5 m-PFOS	0.062 (− 0.050, 0.174)	**0.282 (0.034, 0.531)**	0.005 (− 0.055, 0.064)	0.001 (− 0.125, 0.127)	− 0.006 (− 0.106, 0.095)	− 0.056 (− 0.190, 0.078)	0.097 (− 0.054, 0.248)	− 0.004 (− 0.216, 0.209)	0.013 (− 0.043, 0.068)
Br-PFOS	0.124 (− 0.015, 0.263)	0.178 (− 0.153, 0.508)	0.009 (− 0.066, 0.083)	0.041 (− 0.116, 0.198)	− 0.024 (− 0.149, 0.101)	− 0.091 (− 0.257, 0.075)	0.043 (− 0.145, 0.232)	0.041 (− 0.224, 0.306)	0.017 (− 0.053, 0.086)
n-PFOS	− 0.017 (− 0.142, 0.108)	**0.354 (0.029, 0.678)**	0.011 (− 0.056, 0.077)	0.081 (− 0.059, 0.221)	− 0.058 (− 0.169, 0.053)	− **0.175 (**− **0.322, **− **0.028)**	− 0.109 (− 0.281, 0.063)	− 0.063 (− 0.300, 0.173)	0.042 (− 0.020, 0.103)
6:2 Cl-PFESA	0.025 (− 0.069, 0.119)	0.060 (− 0.175, 0.294)	− 0.043 (− 0.092, 0.007)	− 0.052 (− 0.158, 0.053)	− 0.023 (− 0.107, 0.061)	− **0.228 (**− **0.336, **− **0.120)**	− 0.089 (− 0.217, 0.039)	− 0.008 (− 0.185, 0.169)	0.041 (− 0.006, 0.087)
8:2 Cl-PFESA	− 0.067 (− 0.251, 0.116)	− 0.052 (− 0.507, 0.403)	− 0.065 (− 0.162, 0.033)	− 0.048 (− 0.254, 0.158)	− 0.153 (− 0.315, 0.010)	− 0.196 (− 0.413, 0.021)	− **0.285 (**− **0.527, **− **0.042)**	− 0.209 (− 0.555, 0.137)	0.076 (− 0.015, 0.166)
HFPO-DA	0.023 (− 0.072, 0.118)	0.110 (− 0.106, 0.326)	0.040 (− 0.010, 0.091)	− 0.032 (− 0.139, 0.075)	− 0.020 (− 0.105, 0.066)	− 0.034 (− 0.148, 0.079)	0.002 (− 0.127, 0.131)	− 0.056 (− 0.235, 0.123)	0.024 (− 0.023, 0.071)
PFOA	− 0.012 (− 0.117, 0.093)	0.048 (− 0.216, 0.313)	− 0.012 (− 0.067, 0.044)	− **0.132 (**− **0.249, **− **0.015)**	− 0.084 (− 0.177, 0.010)	− **0.153 (**− **0.277, **− **0.030)**	0.028 (− 0.117, 0.173)	− **0.246 (**− **0.439, **− **0.052)**	**0.060 (0.008, 0.111)**
PFOS	0.034 (− 0.105, 0.174)	**0.364 (0.005, 0.723)**	0.008 (− 0.066, 0.082)	0.079 (− 0.077, 0.235)	− 0.046 (− 0.170, 0.079)	− **0.174 (**− **0.339, **− **0.010)**	− 0.080 (− 0.271, 0.110)	− 0.017 (− 0.281, 0.246)	0.044 (− 0.025, 0.113)

*Adjusted for age, BMI, age at menarche, and education.

Bold characters indicate significance, *P* < 0.05.

### Qgcomp and BKMR analyses

**Figure 1:**
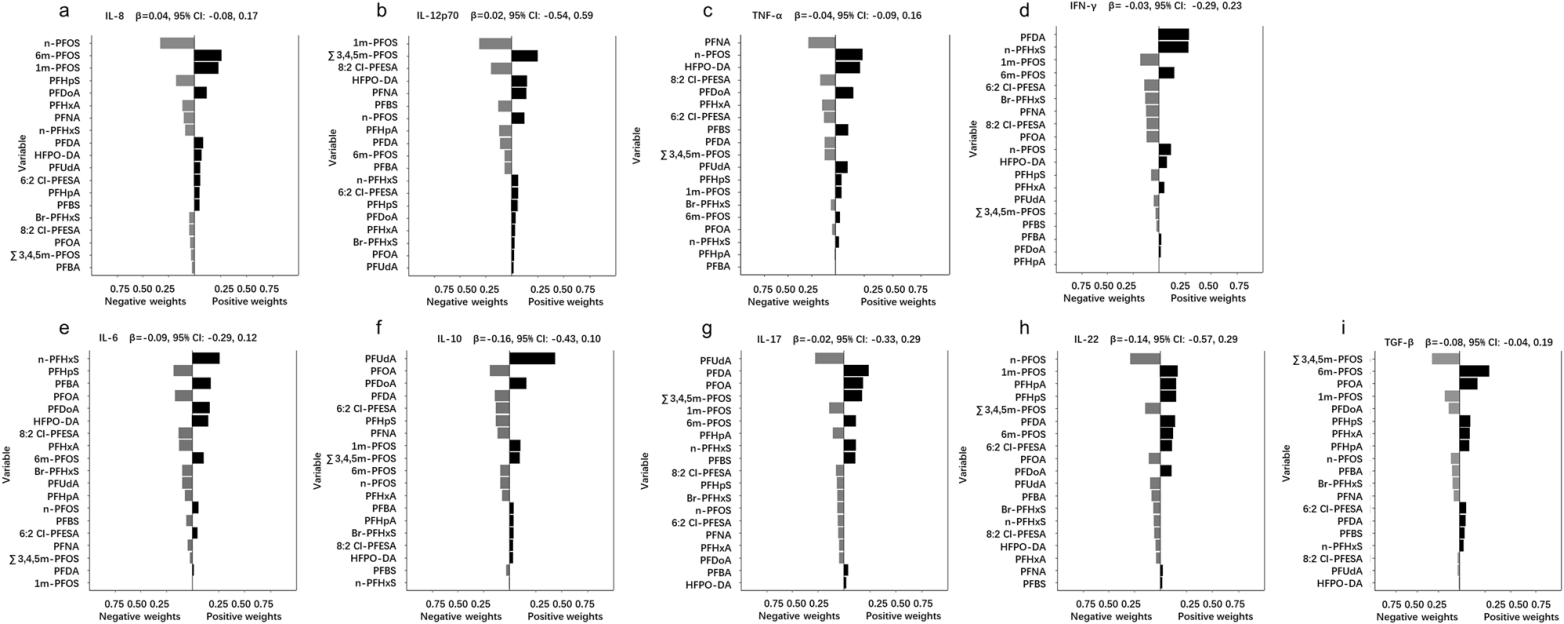
Quantile g-computation regression analysis of the relationship between PFAS levels and cytokines. Models were adjusted for age, BMI, age at menarche, and education.

Figure 1 shows the overall association of the PFAS mixture with cytokines in qgcomp analysis. For Th1 cytokines, 6 m-PFOS (26.1%), ∑3,4,5 m-PFOS (25.2%), n-PFOS (23.8%), and PFDA (29.1%) had the greatest positive contribution to the IL-8, IL-12, TNF-α, and IFN-γ respectively. Also, PFUdA (27.9%) and n-PFOS (29.0%) were the major negative contributors to IL-17 and IL-22, respectively. PFHpS (17.9%) and PFOA (19.1%) had the largest negative weight for IL-6 and IL-10. 6 m-PFOS (29.5%) was the major positive contributor to TGF-β.

**Figure 2:**
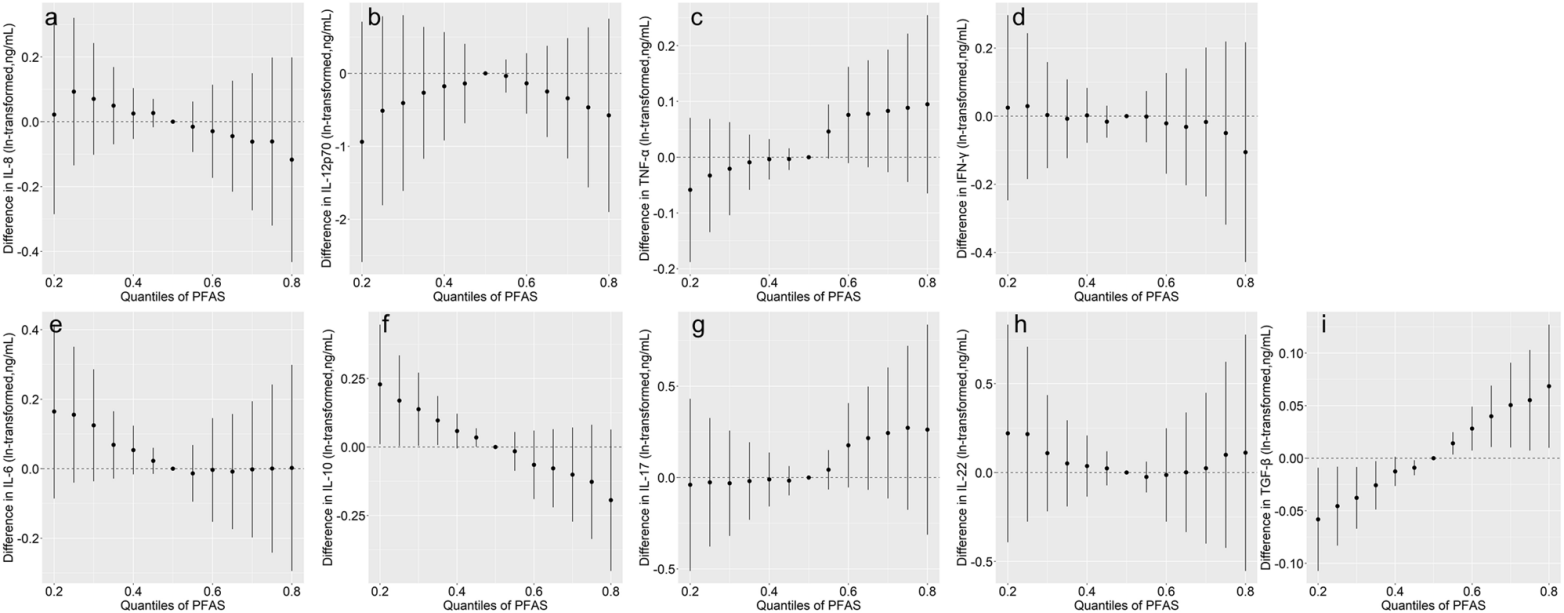
Combined effects of PFAS mixture on changes in ln-cytokines were estimated by Bayesian Kernel Machine Regression (BKMR) model when all PFAS were fixed at a specific percentile compared to when all of them were fixed at the 50th percentile. Models were adjusted for age, BMI, menarche, and education.

In the PFAS mixture analysis, Th1 cytokines have inconsistent trends (Fig. 2a–d), and Th2 cytokines seem to have downward trends (Fig. 2e,f) while IL-17 has an upward trend (Fig. 2g) and IL-22 has a “U” trend (Fig. 2h) but none of them reached statistical significance. Additionally, the increasing levels of PFAS mixture was significantly associated with a higher level of TGF-β when all PFAS were above 55th percentile compared to their 50th percentile (Fig. 2i). PIPs indicated that 1 m-PFOS substantially influenced IL-8 (PIP = 0.454) and IL-12p70 (PIP = 0.896) levels, and 8:2 Cl-PFESA was the primary relevant toxic chemical that contributed to the TNF-α (PIP = 0.218). In addition, for Th2 cytokines, PFHpA (PIP = 0.432) and PFOA (PIP = 0.513) significantly impacted IL-6 and IL-10 levels, respectively. The PIPs also indicated that 6:2 Cl-PFESA (PIP = 0.389) had the highest PIP with IL-17.

## Discussion

Our study found that both legacy and emerging PFAS alternatives were associated with cytokine levels in women of childbearing age. Certain PFAS alternatives and isomers were positively associated with Th1 and Treg cytokines and negatively associated with Th2 and Th17 cytokines.

Consistent with our study, previous studies also reported high concentrations of PFAS alternatives. For example, the plasma concentration of 6:2 Cl-PFESA concentration was 1.4 ng/mL in our study, which is comparable to 1.75 ng/mL reported by Yu et al.^21^ in the general population and 1.54 ng/mL by Chen et al.^22^ in pregnant women in China. Moreover, 6:2 Cl-PFESA, 8:2 Cl-PFESA and HFPO-DA were detected in more than 95% of our population, suggesting that humans are also widely exposed to the PFAS alternatives.

Our results reported that PFAS were positively associated with Th1 cytokines and negatively with Th2 cytokines. Th1 cytokines tend to induce proinflammatory responses while Th2 cells produce anti-inflammatory cytokines. Th1/Th2 balance is beneficial to the maternal–fetal immune tolerance and maintenance of pregnancy. A shift in the Th1/Th2 balance toward Th1 dominance may cause miscarriages^13,14^, preeclampsia^15^, and fetal growth restriction^16^. In addition, this study found a positive association between PFAS and TGF-β. As the principal function of TGF-β is to regulate cellular functions including cell adhesion, invasion and angiogenesis, which is essential for endometriosis development^23^, elevated TGF-β may have effects on endometriosis.

A recent study found that PFHpS was positively associated with IL-8 [β = 0.26, (95% CI: 0.05, 0.48)] in 87 middle-aged women^24^. A significantly inverse association with PFOA was also found for IL-10 (β = − 0.22) among 101 healthy 1-year-old children^25^. Additionally, prenatal PFOS and PFNA in children aged 8 years were inversely related to Th2 cytokines (IL-4 and IL-13) in children from 1,101 mothers-child pairs in the Human Early Life Exposome project (HELIX)^26^. These evidences seem to suggest that PFAS may induce the production of pro-inflammatory cytokines. However, Zhu et al.^27^ showed that serum PFAS were positively associated with Th2 cytokines (IL-4 and IL-5) and negatively associated with Th1 cytokines (IFN-γ and IL-2) among male asthmatics. Positive associations were also observed for PFAS and Th2 cytokine (IL-6) (β = 20.87, 95% CI: 3.46, 41.22) in 103 overweight or obese pregnant women in the U.S.^28^. Thus, evidence so far has been inconsistent.

A number of epidemiological studies in humans focused on the associations between legacy PFAS and immune outcomes such as asthma, allergies, and propensity to infections^29–35^. Dong et al. (2013) reported positive associations between PFOA, PFOS, PFBS, PFDA, PFDoA, PFHxS as well as PFNA exposure and asthma among 231 Taiwanese Children (10–15 years old). They also showed that PFAS were positively associated with serum IgE concentrations, absolute eosinophil counts (AEC), eosinophilic cationic protein (ECP) concentrations, and asthma severity scores among asthmatics^31^. However, PFAS were not statistically associated with the asthma diagnosis, or eosinophil count in 118 World Trade Center (WTC) participants^33^. As for immune globulin, no association between PFOS, PFOA and immunoglobulins IgA-IgG-IgM was found among 53 American male workers in PFOA production department^29^. No significant relationships between serum PFOA and white cell blood counts was discovered in 371 American residents^30^.

Knudsen et al. (2018) reported significant negative associations between PFAS and lymphocyte (β = − 0.3, 95% CI: − 0.47, − 0.13), neutrophil (β = − 0.21, 95% CI: − 0.39, − 0.04), monocyte (β = − 0.29, 95% CI: − 0.46, − 0.13), and white cell counts (β = − 1.56, 95% CI: − 2.41, − 0.70) in 192 Greenlandic pregnant women^32^. Moreover, a longitudinal study found that the PFAS concentrations in 5-year-old children were associated with higher basophil counts (β = 0.46, 95% CI: 0.13, 0.79), but no significant relationship for neutrophils, eosinophils, lymphocytes, monocytes, and white cell counts. Furthermore, no association was found between PFAS from mothers as well as children at 18 months with aforementioned cell counts^34^.

Many in vivo and in vitro experiments have demonstrated that dysregulation of cytokine caused by environmental pollutants induces the inflammatory response^11,36,37^. For example, 10 mg/kg PFOS exposure was positively associated with the expression levels of IL-1β and TNF-α in rats after 14-day exposure^36^. PFAS can also induce a markable upregulation of proinflammatory cytokines TNF-α expression in the jejunum of the PFOS 1 mg/kg and 10 mg/kg groups compared with the control in rats^37^. Collectively, these results indicated that PFOS exposure induces an upregulated pro-inflammatory infiltration. But inconsistent results also reported that PFOS exposure decreased TNF-α and increased IL-10 in the mice colon tissues^38^. Similarly, another study found that exposure to PFOS and F-53B significantly downregulated the expression of pro-inflammatory cytokine genes, including IL-8 and TNF-α, in the liver of adult zebrafish^11^.

The main strength of our study is that we measured various types of PFAS, including PFAS alternatives and isomers. We also measured an array of cytokines including Th1, Th2, Th17, and Treg cytokines. We further used more advanced statistical methods to take into account of PFAS mixture exposure. Thus, our study has given a comprehensive and robust assessment of the association between PFAS, especially the emerging alternatives, and cytokine homeostasis in humans.

Our study also has several limitations. Firstly, the cross-sectional study design does not allow us to draw causal conclusions. Secondly, previous studies showed that other persistent chemicals like polybrominated diphenyl ethers (PBDEs), organochlorine pesticides (OCPs) and bisphenols are also associated with cytokines^28,39^. It remains to be tested whether our finding is due to such unmeasured confounding.

In conclusion, our study suggests that both legacy PFAS and emerging alternatives may affect the homeostasis of cytokines in humans.

## Methods and materials

### Study population

A total of 198 healthy females were recruited in the Center for Reproductive Medicine of Shandong University, China, between 2015 and 2016. Non-pregnant women who had no chronic diseases, endocrine disorders, autoimmune diseases, or active infection were eligible. Blood specimens were collected at the recruitment and centrifuged at 1500 rpm for 20 min to collect serum and plasma, and subsequently stored at − 80 °C until analysis. The study was performed in accordance with the Declaration of Helsinki, the ethics committee of Xinhua Hospital affiliated to Shanghai Jiao Tong University School of Medicine granted ethical clearance for this study (XHEC-C-2015-046), and all participants provided a written informed consent.

### Measurement of PFAS exposure

A total of 21 PFAS analytes were detected in 0.1 mL of plasma by ultra-performance liquid chromatography Agilent 1290 Infinity II coupled with an Agilent 6495C triple-quadrupole tandem mass spectrometry (Agilent Technologies, Palo Alto, CA, USA) in the negative electrospray ionization (ESI-) mode in all subjects. They included PFOA, perfluorononanoic acid (PFNA), perfluorodecanoic acid (PFDA), perfluorododecanoic acid (PFUdA), perfluorododecanoic acid (PFDoA), perfluorohexane sulfonate (PFHpS), 6:2 Cl-PFESA, 8:2 chlorinated perfluoroalkyl ether sulfonic acid (8:2 Cl-PFESA), hexafluoropropylene oxide dimer acid (HFPO-DA)], perfluorobutane sulfonate (PFBS), perfluorobutanoic acid (PFBA), perfluoroheptanoic acid (PFHpA), perfluorohexanoic acid (PFHxA), linear perfluorohexane sulfonate (n-PFHxS), br-PFHxS, linear PFOS (n-PFOS), 1 m-PFOS, 3 m-PFOS, 4 m-PFOS, 5 m-PFOS and 6 m-PFOS. In addition, ∑3,4,5 m-PFOS was calculated as the sum of 3 m-PFOS, 4 m-PFOS, and 5 m-PFOS due to limited separation capacity. Br-PFOS was computed as the sum of 1 m-PFOS, ∑3,4,5 m-PFOS and 6 m-PFOS, and total PFOS (PFOS) was the sum of n-PFOS and br-PFOS. Similarly, total PFHxS (PFHxS) was the sum of n-PFHxS and br-PFHxS. Internal quality control samples and procedure blanks were included along with each batch of samples for quality assurance throughout the project.

Extraction methods were modified by Kuklenyik et al.^40^ and Benskin et al.^41^. Briefly, the Oasis HLB extraction cartridges (3 cc) (Waters Corp., Milford, MA, USA) were pretreated with 1 mL methanol and 1 mL formic acid (0.1 M). 100 μL plasma samples were spiked with 2 ng of mass-labelled standard, and 1 mL formic acid (0.1 M). After shaking, the sample was loaded onto the cartridge. Then the cartridge was washed with 1 mL 0.1 M formic acid, 3 mL of 50% 0.1 M formic acid/50% methanol, and 0.5 mL of 1% ammonium hydroxide in succession. The PFAS were eluted with 1.8 mL of 1% ammonium hydroxide in acetonitrile (ACN). The eluate was evaporated and reconstituted to 0.1 mL in a mixture of methanol and 10 mM ammonium acetate. Finally, the mixture was transferred to UPLC-MS/MS for analysis. The limit of detection (LOD) was from 0.002 to 0.0083 ng/mL for all PFAS examined. More detailed information on LOD and limits of quantification (LOQ) can be found in Supplementary Table S1. Measurements below the LODs were replaced by LOD divided by the square root of 2^42^.

### Outcome assessment

The method for measuring cytokines in serum has been detailed in a previous study^43^. Briefly, cytokines including Th1 cytokines (IL-1β, IL-2, IL-8, IL-12, IL-13, TNF-α and IFN-γ), Th2 cytokines (IL-4, IL-6 and IL-10), and Th17 cytokines (IL-17 and IL-22) were quantified by a multiplex assay U-PLEX platform with electrochemiluminescent enzyme-linked immunosorbent assays [Meso Scale Discovery (MSD), Rockville, Maryland, USA]. And Treg cytokines (TGF-β) were measured by a single-plex assay V-PLEX assay platform following the manufacturer’s instructions. MESO QuickPlex SQ 120 plate reader [Meso Scale Discovery (MSD), Rockville, Maryland, USA] was used to determine the analyte concentration.

### Covariates

Potential confounding factors included age (year, continuous), age at menarche (year, continuous), body mass index (BMI, kg/m^2^, continuous), and education (primary, middle, and above middle school).

### Statistical analysis

The descriptive statistics were performed as mean ± standard deviation (SD) or number (percentage) for general characteristics among the participants. The plasma PFAS concentrations and serum cytokines were presented as median (interquartile range) and mean (SD). PFAS and cytokines concentrations were natural log transformed to reduce skewness in further analyses. Spearman’s rank correlation was used to assess correlations between different plasma PFAS.

Multiple linear regression models were used to assess the associations between plasma PFAS concentrations and cytokines. In adjusted single-PFAS models, age, age at menarche, BMI, and education were used as covariates. Subsequently, PFAS levels were categorized into quartiles and the β coefficients (95% CI) of cytokine were estimated using the first quartile as the reference.

Quantile g-computation (gqcomp) is an improved modeling method based on weighted quantile sum (WQS) regression that can assess the joint effect of all mixture exposures by one quartile at a time^44^. Individual exposure-outcome relationships in the mixture can be inconsistent, and this method produces unbiased estimates of overall mixture effects in small sample sizes with an acceptable CI coverage^44^. It has the advantage to allow chemicals to interact with the outcome in either a positive or negative direction and is performed in the following three steps. First, all PFAS were categorized into quantiles. Second, PFAS, covariates, and cytokines were fit in a linear model. Finally, an index weight was calculated between 0 and 1 for each exposure, corresponding to the contributing individual component of the mixture effect on the outcome. The overall mixture effect can be considered as the change in outcome per one quantile of change in all PFAS exposures after adjusting for covariates.

Bayesian kernel machine regression (BKMR)^45^ was conducted as a semi-parametric machine learning method to investigate non-linear dose–response associations, interactions among PFAS, and overall cumulative effects of the PFAS mixture. In this study, the BKMR models were applied as Y_i_ = h (PFAS) + ß^T^ Z_i_ + e_i_, where Y_i_ is the change of cytokines; h () is an exposure–response kernel function; Z_i_ contains a set of potential confounders; β is the regression coefficient; and e_i_ represents residuals. We identified the most important PFAS contributing to cytokine changes within the mixture using the hierarchical variable selection and presented the posterior inclusion probability (PIP) for each PFAS.

Most statistical analyses were performed using the survey analysis procedures in SAS (version 9.4; SAS Institute Inc., Cary, NC, USA). In addition, we used R software (version 4.0.3; R foundation for Statistical Computing, Vienna, Austria) with “qgcomp” and “bkmr” packages to conduct PFAS mixture analyses. All the *P*-values were two-tailed, and statistical significance was defined as p value less than 0.05.

## Supplementary Information

Supplementary Information.

## Data Availability

The datasets generated and analyzed during the current study are not publicly available due to participant confidentiality but are available from the corresponding author on reasonable request.
