# Supplementary material for: Emerging and legacy PFAS and cytokine homeostasis in women of childbearing age
Source: Sci Rep. 2022 Apr 20;12:6517. doi: 10.1038/s41598-022-10501-8 (PMC9021217; doi:10.1038/s41598-022-10501-8)
Supplement: Supplementary file 1 — Supplementary Information. [file 41598_2022_10501_MOESM1_ESM.pdf]

Supplemental information

Title: Emerging and Legacy PFAS and Cytokine Homeostasis in Women of Childbearing Age

Authors: Min Nian, Wei Zhou, Yan Feng, Yan Wang, Qian Chen, Jun Zhang

Table S1. Names, acronyms and detection limit of target perfluorinated chemicals

Table S2. Associations between single ln-transformed PFAS and cytokines in multivariable linear regression (n=198)

Table S3. Linear associations of PFAS isomers (divided in quantiles) with Th1 and Th2 cytokines concentrations (ln-transformed) (n=198)

Table S4. Linear associations of PFAS isomers (divided in quantiles) with Th17 and Treg cytokines concentrations (ln-transformed) (n=198)

Table S5. Linear associations of PFAS alternatives (divided in quantiles) with cytokines concentrations (ln-transformed) (n=198)

Table S6. Linear associations of short-chain PFAS (divided in quantiles) with cytokines concentrations (ln-transformed) (n=198)

Table S7. Linear associations of legacy PFAS (divided in quantiles) with Th1 and Th2 cytokines concentrations (ln-transformed) (n=198)

Table S8. Linear associations of PFAS isomers (divided in quantiles) with Th17 and Treg cytokines concentrations (ln-transformed) (n=198)

Fig. S1. Correlations between plasma concentrations of PFAS.

Table S1. Names, acronyms and detection limit of target perfluorinated chemicals

Acronym	Chemical full name	Quantification transitions	Surrogate standards	LOD (ng/mL)
PFHxS	Perfluorohexane sulfonate	/	/	/
PFOA	Perfluorooctanoic acid	413/369	M8PFOA	0.0083
PFHpS	Perfluoroheptane sulfonate	449/80	M3PFHxS	0.0051
PFNA	Perfluorononanoic acid	463/419	M9PFNA	0.0037
PFOS	Perfluorooctane sulfonate	/	/	/
PFDA	Perfluorodecanoic acid	513/469	M6PFDA	0.0017
PFUdA	Perfluoroundecanoic acid	563/519	M7PFUdA	0.0072
PFDoA	Perfluorododecanoic acid	613/569	MPFDoA	0.0038
n-PFHxS	Linear perfluorohexane	399/80	M3PFHxS	0.0066
Br-PFHxS	Sum of all branched isomers	399/80	M3PFHxS	0.0025
n-PFOS	Linear perfluorooctane sulfonate	499/80	M8PFOS	0.0044
1m-PFOS	1-perfluorooctane sulfonate	499/419	M8PFOS	0.0044
6m-PFOS	6-perfluorooctane sulfonate	499/80	M8PFOS	0.0013
∑3,4,5m-PFOS	Sum of all 3,4,5-perfluorooctane sulfonate	499/80	M8PFOS	0.0005
Br-PFOS	Sum of all branched isomers	/	/	/
HFPO-DA	Hexafluoropropylene oxide dimer acid	285/185	M8PFOA	0.0064
6:2 Cl-PFESA	6:2 chlorinated perfluoroalkyl ether sulfonic acid	531/361	M8PFOS	0.0027
8:2 Cl-PFESA	8:2 chlorinated perfluoroalkyl ether sulfonic acid	631/451	M8PFOS	0.0054
PFBA	Perfluoro-n-butanoic acid	213/169	MPFBA	0.006
PFBS	Perfluorobutane sulfonate	299/80	M3PFBS	0.003
PFHxA	Perfluorohexanoic acid	313/269	M5PFHxA	0.002
PFHpA	Perfluoroheptanoic acid	363/319	M4PFHpA	0.002

Table S2. Associations between single ln-transformed PFAS and cytokines in multivariable linear regression (n=198)*

PFAS	Th1				Th2		Th17		Treg
	IL-8	IL-12p70	TNF-α	IFN-γ	IL-6	IL-10	IL-17	IL-22	TGF-β
PFBS	0.062 (-0.023, 0.146)	-0.048 (-0.247, 0.150)	0.023 (-0.022, 0.069)	-0.011 (-0.107, 0.085)	-0.023 (-0.099, 0.053)	-0.034 (-0.136, 0.067)	0.030 (-0.086, 0.146)	0.114 (-0.049, 0.278)	0.014 (-0.028, 0.056)
PFHpA	-0.014 (-0.101, 0.073)	-0.163 (-0.389, 0.063)	0.000 (-0.046, 0.046)	-0.050 (-0.147, 0.047)	-0.062 (-0.139, 0.016)	-0.010 (-0.113, 0.094)	-0.057 (-0.172, 0.059)	0.059 (-0.109, 0.227)	0.038 (-0.005, 0.081)
PFBA	0.065 (-0.025, 0.155)	0.015 (-0.175, 0.205)	0.018 (-0.030, 0.066)	-0.083 (-0.184, 0.018)	-0.011 (-0.092, 0.070)	0.037 (-0.071, 0.145)	-0.031 (-0.151, 0.088)	-0.007 (-0.179, 0.164)	-0.005 (-0.050, 0.040)
PFHxA	-0.035 (-0.110, 0.041)	-0.033 (-0.211, 0.145)	-0.011 (-0.051, 0.029)	-0.023 (-0.108, 0.063)	**-0.086 (-0.152, -0.019)**	-0.069 (-0.158, 0.021)	-0.076 (-0.177, 0.025)	-0.059 (-0.202, 0.084)	0.018 (-0.019, 0.055)
PFNA	0.033 (-0.108, 0.174)	0.286 (-0.051, 0.624)	0.009 (-0.066, 0.084)	0.006 (-0.153, 0.164)	-0.067 (-0.193, 0.059)	**-0.182 (-0.348, -0.015)**	-0.143 (-0.333, 0.047)	-0.081 (-0.345, 0.182)	**0.076 (0.006, 0.145)**
PFDA	0.059 (-0.064, 0.182)	0.273 (-0.058, 0.604)	-0.004 (-0.070, 0.061)	0.096 (-0.042, 0.234)	-0.041 (-0.151, 0.069)	-0.121 (-0.267, 0.026)	-0.144 (-0.311, 0.024)	-0.039 (-0.271, 0.194)	0.036 (-0.025, 0.097)
PFHxS	-0.054 (-0.212, 0.104)	0.307 (-0.070, 0.683)	0.029 (-0.054, 0.113)	0.144 (-0.033, 0.320)	0.064 (-0.077, 0.205)	-0.182 (-0.369, 0.004)	0.025 (-0.186, 0.236)	0.056 (-0.248, 0.361)	**0.082 (0.004, 0.160)**
PFHpS	0.123 (-0.074, 0.319)	0.378 (-0.113, 0.869)	0.092 (-0.012, 0.196)	-0.020 (-0.242, 0.201)	-0.011 (-0.187, 0.165)	-0.216 (-0.449, 0.017)	-0.155 (-0.424, 0.113)	0.023 (-0.357, 0.404)	**0.097 (0.000, 0.194)**
PFDoA	**0.123 (0.010, 0.236)**	-0.134 (-0.360, 0.091)	0.028 (-0.032, 0.089)	0.014 (-0.114, 0.143)	-0.008 (-0.111, 0.094)	0.124 (-0.011, 0.260)	-0.098 (-0.251, 0.054)	0.169 (-0.046, 0.384)	-0.025 (-0.082, 0.032)
PFUdA	-0.007 (-0.154, 0.140)	**0.375 (0.043, 0.707)**	0.014 (-0.064, 0.092)	0.026 (-0.139, 0.192)	-0.073 (-0.204, 0.059)	-0.114 (-0.289, 0.061)	-0.193 (-0.391, 0.005)	-0.085 (-0.361, 0.190)	0.069 (-0.004, 0.142)

* Adjusted for age, BMI, age at menarche, and education.

Bold characters indicate significance, $P < 0.05$.

Table S3. Linear associations of PFAS isomers (divided in quantiles) with Th1 and Th2 cytokines concentrations (ln-transformed) (n=198)*.

PFAS isomers	Th1				Th2	
	IL-8	IL-12p70	TNF-α	IFN-γ	IL-6	IL-10
n-PFHxS						
Q1	Ref	Ref	Ref	Ref	Ref	Ref
Q2	0.24 (-0.05, 0.52)	0.46 (-0.28, 1.20)	0.08 (-0.07, 0.23)	0.28 (-0.04, 0.60)	0.17 (-0.08, 0.43)	0.09 (-0.25, 0.43)
Q3	0.11 (-0.18, 0.40)	0.48 (-0.31, 1.27)	-0.05 (-0.20, 0.11)	0.01 (-0.32, 0.33)	-0.04 (-0.30, 0.22)	-0.08 (-0.43, 0.27)
Q4	0.07 (-0.22, 0.35)	0.57 (-0.16, 1.31)	0.05 (-0.10, 0.20)	0.28 (-0.04, 0.60)	0.14 (-0.11, 0.40)	-0.21 (-0.56, 0.13)
P-trend	0.872	0.177	0.914	0.267	0.592	0.140
Br-PFHxS						
Q1	Ref	Ref	Ref	Ref	Ref	Ref
Q2	-0.24 (-0.53, 0.05)	-0.17 (-0.97, 0.63)	0.05 (-0.10, 0.20)	0.08 (-0.25, 0.41)	0.05 (-0.21, 0.31)	-0.24 (-0.59, 0.11)
Q3	0.02 (-0.25, 0.29)	0.11 (-0.52, 0.74)	0.06 (-0.08, 0.21)	-0.09 (-0.40, 0.22)	-0.01 (-0.26, 0.23)	-0.11 (-0.44, 0.21)
Q4	-0.17 (-0.43, 0.10)	0.34 (-0.37, 1.05)	-0.06 (-0.20, 0.08)	-0.06 (-0.36, 0.25)	-0.17 (-0.41, 0.07)	-0.22 (-0.54, 0.10)
P-trend	0.487	0.327	0.552	0.541	0.169	0.258
n-PFOS						
Q1	Ref	Ref	Ref	Ref	Ref	Ref
Q2	-0.02 (-0.31, 0.27)	-0.41 (-1.15, 0.32)	0.01 (-0.14, 0.16)	0.01 (-0.31, 0.33)	-0.25 (-0.50, 0.01)	-0.13 (-0.47, 0.21)
Q3	-0.06 (-0.35, 0.23)	0.35 (-0.36, 1.05)	0.00 (-0.16, 0.15)	0.05 (-0.28, 0.37)	-0.24 (-0.50, 0.02)	-0.15 (-0.48, 0.19)
Q4	0.02 (-0.27, 0.31)	0.47 (-0.24, 1.18)	0.06 (-0.10, 0.21)	0.11 (-0.21, 0.43)	-0.09 (-0.35, 0.16)	**-0.47 (-0.81, -0.13)**
P-trend	0.960	**0.036**	0.515	0.479	0.533	**0.009**
6m-PFOS						
Q1	Ref	Ref	Ref	Ref	Ref	Ref
Q2	-0.04 (-0.33, 0.24)	0.44 (-0.29, 1.17)	-0.04 (-0.19, 0.12)	-0.02 (-0.34, 0.31)	-0.21 (-0.46, 0.05)	-0.13 (-0.46, 0.21)
Q3	0.08 (-0.21, 0.37)	0.45 (-0.29, 1.19)	0.01 (-0.14, 0.17)	0.04 (-0.28, 0.37)	-0.07 (-0.32, 0.19)	0.03 (-0.31, 0.37)
Q4	0.25 (-0.04, 0.53)	0.56 (-0.14, 1.26)	-0.01 (-0.16, 0.14)	0.06 (-0.26, 0.38)	-0.05 (-0.31, 0.20)	**-0.41 (-0.75, -0.08)**
P-trend	0.059	0.145	0.973	0.651	0.957	**0.04**
1m-PFOS						
Q1	Ref	Ref	Ref	Ref	Ref	Ref
Q2	-0.02 (-0.30, 0.26)	0.38 (-0.38, 1.15)	-0.02 (-0.17, 0.13)	-0.10 (-0.42, 0.22)	-0.21 (-0.46, 0.04)	0.15 (-0.19, 0.49)
Q3	0.12 (-0.17, 0.40)	0.16 (-0.54, 0.87)	0.04 (-0.12, 0.19)	-0.01 (-0.34, 0.32)	0.00 (-0.25, 0.26)	0.13 (-0.21, 0.48)
Q4	**0.30 (0.02, 0.58)**	-0.02 (-0.69, 0.64)	0.03 (-0.12, 0.18)	-0.08 (-0.40, 0.24)	-0.06 (-0.31, 0.20)	-0.18 (-0.52, 0.15)
P-trend	**0.021**	0.838	0.550	0.738	0.948	0.292
∑3,4,5m-PFOS						
Q1	Ref	Ref	Ref	Ref	Ref	Ref
Q2	0.00 (-0.28, 0.28)	0.25 (-0.47, 0.98)	-0.03 (-0.18, 0.12)	-0.05 (-0.37, 0.27)	-0.05 (-0.30, 0.21)	0.00 (-0.34, 0.34)
Q3	0.05 (-0.23, 0.34)	0.40 (-0.30, 1.10)	-0.02 (-0.17, 0.14)	-0.17 (-0.50, 0.15)	0.02 (-0.23, 0.28)	0.03 (-0.31, 0.37)
Q4	0.25 (-0.03, 0.53)	0.39 (-0.27, 1.06)	0.00 (-0.16, 0.15)	0.03 (-0.29, 0.35)	-0.03 (-0.29, 0.23)	-0.10 (-0.44, 0.24)
P-trend	0.076	0.225	0.990	0.978	0.960	0.614
Total br-PFOS						

Q1	Ref	Ref	Ref	Ref	Ref	Ref
Q2	-0.03 (-0.31, 0.25)	0.31 (-0.47, 1.10)	-0.11 (-0.26, 0.04)	0.00 (-0.32, 0.32)	-0.01 (-0.27, 0.24)	-0.15 (-0.49, 0.19)
Q3	0.09 (-0.20, 0.38)	0.42 (-0.25, 1.09)	-0.01 (-0.16, 0.14)	-0.10 (-0.42, 0.23)	0.08 (-0.17, 0.34)	-0.05 (-0.40, 0.29)
Q4	0.23 (-0.06, 0.51)	0.40 (-0.26, 1.06)	-0.01 (-0.17, 0.14)	0.10 (-0.22, 0.42)	-0.03 (-0.29, 0.22)	-0.21 (-0.55, 0.13)
P-trend	0.08	0.210	0.803	0.680	0.994	0.332

[*] Adjusted for age, BMI, age at menarche, and education.

Bold characters indicate significance, $P < 0.05$.

Table S4. Linear associations of PFAS isomers (divided in quantiles) with Th17 and Treg cytokines concentrations (ln-transformed) (n=198)*.

PFAS isomers	Th17		Treg
	IL-17	IL-22	TGF-β
n-PFHxS			
Q1	Ref	Ref	Ref
Q2	0.37 (-0.01, 0.76)	0.25 (-0.30, 0.79)	0.05 (-0.09, 0.20)
Q3	0.28 (-0.11, 0.67)	-0.48 (-1.02, 0.07)	0.08 (-0.06, 0.23)
Q4	0.14 (-0.24, 0.53)	0.03 (-0.51, 0.56)	0.10 (-0.05, 0.24)
P-trend	0.598	0.501	0.172
Br-PFHxS			
Q1	Ref	Ref	Ref
Q2	-0.08 (-0.47, 0.32)	0.09 (-0.47, 0.65)	0.08 (-0.06, 0.23)
Q3	0.16 (-0.21, 0.53)	-0.02 (-0.54, 0.49)	0.06 (-0.07, 0.20)
Q4	-0.02 (-0.38, 0.34)	-0.39 (-0.90, 0.11)	0.05 (-0.08, 0.18)
P-trend	0.799	0.139	0.491
n-PFOS			
Q1	Ref	Ref	Ref
Q2	-0.23 (-0.62, 0.16)	-0.29 (-0.83, 0.25)	-0.01 (-0.16, 0.13)
Q3	-0.16 (-0.55, 0.23)	-0.26 (-0.81, 0.29)	0.08 (-0.07, 0.22)
Q4	-0.32 (-0.71, 0.07)	-0.29 (-0.83, 0.25)	0.05 (-0.09, 0.19)
P-trend	0.158	0.342	0.284
6m-PFOS			
Q1	Ref	Ref	Ref
Q2	0.34 (-0.05, 0.72)	-0.22 (-0.76, 0.33)	0.09 (-0.05, 0.23)
Q3	0.31 (-0.09, 0.70)	0.03 (-0.53, 0.59)	0.09 (-0.06, 0.23)
Q4	0.14 (-0.25, 0.52)	-0.04 (-0.57, 0.50)	0.10 (-0.04, 0.24)
P-trend	0.576	0.894	0.209
1m-PFOS			
Q1	Ref	Ref	Ref
Q2	-0.10 (-0.48, 0.29)	-0.12 (-0.65, 0.41)	-0.01 (-0.15, 0.14)
Q3	0.08 (-0.31, 0.47)	-0.35 (-0.89, 0.20)	0.08 (-0.06, 0.23)
Q4	-0.01 (-0.40, 0.37)	0.24 (-0.29, 0.78)	0.00 (-0.14, 0.14)
P-trend	0.847	0.548	0.684
∑3,4,5m-PFOS			
Q1	Ref	Ref	Ref
Q2	0.25 (-0.13, 0.63)	-0.40 (-0.93, 0.14)	0.13 (-0.01, 0.27)
Q3	0.32 (-0.06, 0.71)	**-0.56 (-1.10, -0.03)**	0.05 (-0.09, 0.20)
Q4	0.28 (-0.10, 0.66)	-0.03 (-0.56, 0.50)	0.00 (-0.14, 0.14)
P-trend	0.146	0.767	0.746
Total br-PFOS			
Q1	Ref	Ref	Ref
Q2	-0.09 (-0.47, 0.28)	-0.30 (-0.83, 0.24)	0.05 (-0.09, 0.19)
Q3	0.20 (-0.19, 0.59)	-0.27 (-0.82, 0.27)	0.13 (-0.02, 0.27)
Q4	0.10 (-0.28, 0.49)	0.10 (-0.43, 0.64)	0.01 (-0.14, 0.15)
P-trend	0.346	0.708	0.713

* Adjusted for age, BMI, age at menarche, and education.

Bold characters indicate significance, $P < 0.05$.

Table S5. Linear associations of PFAS alternatives (divided in quantiles) with cytokines concentrations (ln-transformed) (n=198)*.

PFAS isomers	Th1				Th2		Th17		Treg
	IL-8	IL-12p70	TNF-α	IFN-γ	IL-6	IL-10	IL-17	IL-22	TGF-β
6:2 Cl-PFESA									
Q1	Ref	Ref	Ref	Ref	Ref	Ref	Ref	Ref	Ref
Q2	0.00<br>(-0.29, 0.28)	-0.42<br>(-1.13, 0.30)	-0.06<br>(-0.21, 0.10)	-0.20<br>(-0.52, 0.12)	-0.06<br>(-0.32, 0.20)	**-0.55**<br>**(-0.88, -0.23)**	0.10<br>(-0.30, 0.49)	0.06<br>(-0.48, 0.60)	0.04<br>(-0.10, 0.18)
Q3	0.21<br>(-0.07, 0.50)	0.09<br>(-0.62, 0.80)	0.01<br>(-0.14, 0.17)	-0.29<br>(-0.61, 0.03)	-0.11<br>(-0.37, 0.14)	**-0.47**<br>**(-0.80, -0.14)**	-0.09<br>(-0.48, 0.29)	-0.02<br>(-0.57, 0.52)	0.11<br>(-0.03, 0.26)
Q4	0.07<br>(-0.22, 0.35)	0.13<br>(-0.55, 0.82)	-0.07<br>(-0.22, 0.08)	-0.03<br>(-0.35, 0.28)	-0.04<br>(-0.29, 0.22)	**-0.66**<br>**(-0.98, -0.33)**	-0.25<br>(-0.64, 0.13)	0.12<br>(-0.42, 0.66)	0.13<br>(-0.01, 0.27)
P-trend	0.359	0.409	0.561	0.718	0.695	**<0.001**	0.124	0.759	**0.045**
8:2 Cl-PFESA									
Q1	Ref	Ref	Ref	Ref	Ref	Ref	Ref	Ref	Ref
Q2	0.07<br>(-0.22, 0.35)	0.40<br>(-0.32, 1.13)	**0.19**<br>**(0.04, 0.33)**	0.32<br>(0.01, 0.64)	-0.01<br>(-0.26, 0.25)	0.20<br>(-0.14, 0.53)	-0.01<br>(-0.39, 0.37)	-0.15<br>(-0.68, 0.38)	0.01<br>(-0.13, 0.15)
Q3	-0.11<br>(-0.39, 0.17)	0.29<br>(-0.40, 0.99)	0.12<br>(-0.03, 0.26)	-0.11<br>(-0.43, 0.20)	-0.11<br>(-0.37, 0.14)	0.00<br>(-0.33, 0.34)	-0.16<br>(-0.55, 0.22)	-0.44<br>(-0.97, 0.08)	0.11<br>(-0.03, 0.25)
Q4	0.07<br>(-0.21, 0.35)	-0.31<br>(-1.04, 0.42)	-0.02<br>(-0.17, 0.12)	-0.04<br>(-0.35, 0.28)	-0.18<br>(-0.43, 0.08)	-0.18<br>(-0.52, 0.15)	-0.30<br>(-0.68, 0.08)	-0.34<br>(-0.89, 0.20)	0.04<br>(-0.10, 0.18)
P-trend	0.961	0.378	0.586	0.296	0.120	0.180	0.085	0.121	0.346
HFPO-DA									
Q1	Ref	Ref	Ref	Ref	Ref	Ref	Ref	Ref	Ref
Q2	-0.17<br>(-0.45, 0.12)	-0.55<br>(-1.31, 0.21)	**-0.24**<br>**(-0.39, -0.09)**	-0.24<br>(-0.56, 0.08)	-0.01<br>(-0.27, 0.24)	-0.19<br>(-0.53, 0.15)	-0.04<br>(-0.43, 0.35)	-0.36<br>(-0.89, 0.17)	-0.08<br>(-0.22, 0.07)
Q3	0.14<br>(-0.15, 0.42)	0.05<br>(-0.66, 0.75)	-0.03<br>(-0.17, 0.12)	-0.20<br>(-0.52, 0.12)	0.11<br>(-0.15, 0.36)	-0.10<br>(-0.43, 0.24)	0.03<br>(-0.35, 0.42)	0.01<br>(-0.53, 0.55)	0.02<br>(-0.12, 0.16)
Q4	-0.11<br>(-0.39, 0.18)	0.08<br>(-0.57, 0.74)	0.02<br>(-0.12, 0.17)	-0.10<br>(-0.42, 0.22)	-0.04<br>(-0.30, 0.21)	-0.27<br>(-0.61, 0.07)	-0.17<br>(-0.55, 0.22)	-0.42<br>(-0.96, 0.12)	0.06<br>(-0.08, 0.20)
P-trend	0.964	0.482	0.252	0.642	0.981	0.193	0.493	0.297	0.238

* Adjusted for age, BMI, age at menarche, and education.

Bold characters indicate significance, $P < 0.05$.

Table S6. Linear associations of short-chain PFAS (divided in quantiles) with cytokines concentrations (ln-transformed) (n=198)*.

PFAS isomers	Th1				Th2		Th17		Treg
	IL-8	IL-12p70	TNF-α	IFN-γ	IL-6	IL-10	IL-17	IL-22	TGF-β
PFBS									
Q1	Ref	Ref	Ref	Ref	Ref	Ref	Ref	Ref	Ref
Q2	-0.10 (-0.38, 0.19)	0.25 (-0.42, 0.93)	-0.06 (-0.21, 0.10)	0.01 (-0.31, 0.34)	-0.04 (-0.29, 0.22)	-0.23 (-0.57, 0.11)	0.13 (-0.25, 0.52)	-0.17 (-0.72, 0.39)	0.01 (-0.13, 0.16)
Q3	-0.23 (-0.51, 0.05)	0.55 (-0.12, 1.22)	0.06 (-0.09, 0.21)	0.09 (-0.24, 0.41)	-0.04 (-0.30, 0.21)	0.09 (-0.25, 0.42)	0.30 (-0.08, 0.69)	0.24 (-0.30, 0.79)	0.04 (-0.10, 0.18)
Q4	0.19 (-0.09, 0.47)	-0.53 (-1.15, 0.10)	0.06 (-0.09, 0.21)	-0.09 (-0.41, 0.23)	-0.14 (-0.39, 0.11)	-0.27 (-0.61, 0.06)	0.14 (-0.25, 0.53)	0.17 (-0.37, 0.72)	0.06 (-0.08, 0.20)
P-trend	0.351	0.220	0.229	0.720	0.303	0.363	0.348	0.283	0.388
PFHpA									
Q1									
Q2	-0.15 (-0.43, 0.13)	-0.25 (-0.97, 0.48)	-0.03 (-0.18, 0.12)	0.06 (-0.26, 0.38)	0.00 (-0.25, 0.25)	0.14 (-0.19, 0.47)	-0.23 (-0.61, 0.15)	0.29 (-0.25, 0.83)	0.10 (-0.04, 0.24)
Q3	0.08 (-0.20, 0.37)	-0.36 (-1.09, 0.37)	-0.07 (-0.22, 0.08)	-0.15 (-0.47, 0.17)	0.01 (-0.25, 0.26)	**0.35 (0.02, 0.68)**	0.05 (-0.34, 0.43)	0.31 (-0.23, 0.85)	-0.05 (-0.19, 0.09)
Q4	0.04 (-0.25, 0.32)	-0.34 (-1.05, 0.37)	0.03 (-0.13, 0.18)	0.00 (-0.32, 0.32)	-0.10 (-0.35, 0.16)	-0.09 (-0.42, 0.25)	-0.20 (-0.58, 0.18)	0.48 (-0.06, 1.02)	**0.15 (0.01, 0.29)**
P-trend	0.439	0.335	0.879	0.693	0.486	0.926	0.595	0.098	0.206
PFBA									
Q1									
Q2	**0.32 (0.04, 0.60)**	0.23 (-0.46, 0.92)	0.12 (-0.03, 0.27)	-0.23 (-0.55, 0.09)	0.06 (-0.19, 0.32)	0.06 (-0.28, 0.40)	-0.02 (-0.41, 0.36)	-0.03 (-0.59, 0.52)	0.08 (-0.06, 0.22)
Q3	0.13 (-0.16, 0.42)	-0.26 (-0.94, 0.41)	0.06 (-0.10, 0.21)	-0.20 (-0.53, 0.12)	0.14 (-0.12, 0.41)	0.04 (-0.30, 0.39)	-0.10 (-0.49, 0.30)	0.02 (-0.53, 0.57)	0.03 (-0.11, 0.18)
Q4	0.20 (-0.08, 0.49)	0.00 (-0.76, 0.77)	0.11 (-0.04, 0.26)	-0.10 (-0.42, 0.22)	0.09 (-0.16, 0.35)	0.08 (-0.27, 0.42)	0.01 (-0.38, 0.40)	0.30 (-0.24, 0.84)	0.02 (-0.13, 0.16)
P-trend	0.353	0.683	0.270	0.607	0.394	0.702	0.934	0.273	0.968
PFHxA									
Q1									
Q2	-0.04 (-0.32, 0.25)	-0.23 (-1.01, 0.56)	-0.06 (-0.21, 0.09)	0.10 (-0.23, 0.42)	**-0.32 (-0.58, -0.07)**	0.05 (-0.29, 0.39)	0.26 (-0.12, 0.63)	-0.10 (-0.64, 0.43)	-0.14 (-0.28, 0.00)
Q3	-0.04 (-0.33, 0.25)	-0.44 (-1.10, 0.23)	-0.05 (-0.20, 0.11)	0.19 (-0.13, 0.51)	-0.18 (-0.43, 0.07)	-0.23 (-0.57, 0.11)	-0.23 (-0.61, 0.15)	-0.01 (-0.55, 0.54)	-0.01 (-0.15, 0.13)
Q4	-0.16 (-0.44, 0.13)	-0.02 (-0.69, 0.65)	-0.07 (-0.22, 0.08)	-0.07 (-0.39, 0.25)	**-0.33 (-0.58, -0.08)**	-0.25 (-0.58, 0.09)	0.01 (-0.37, 0.39)	-0.29 (-0.83, 0.25)	0.07 (-0.07, 0.21)

P-trend	0.300	0.782	0.428	0.802	**0.039**	0.058	0.458	0.368	0.122

[*] Adjusted for age, BMI, age at menarche, and education.

Bold characters indicate significance, $P < 0.05$.

Table S7. Linear associations of legacy PFAS (divided in quantiles) with Th1 and Th2 cytokines concentrations (ln-transformed) (n=198)*.

PFAS isomers	Th1				Th2	
	IL-8	IL-12p70	TNF-α	IFN-γ	IL-6	IL-10
PFOA						
Q1	Ref	Ref	Ref	Ref	Ref	Ref
Q2	-0.16 (-0.44, 0.13)	0.35 (-0.38, 1.07)	0.13 (-0.02, 0.28)	-0.24 (-0.56, 0.08)	-0.13 (-0.38, 0.13)	-0.01 (-0.35, 0.32)
Q3	0.05 (-0.23, 0.33)	-0.04 (-0.78, 0.70)	0.12 (-0.03, 0.26)	-0.06 (-0.38, 0.25)	**-0.26 (-0.51, -0.01)**	-0.13 (-0.46, 0.20)
Q4	-0.13 (-0.42, 0.16)	0.51 (-0.22, 1.24)	-0.05 (-0.20, 0.10)	**-0.38 (-0.70, -0.05)**	-0.25 (-0.50, 0.01)	**-0.50 (-0.84, -0.17)**
P-trend	0.697	0.322	0.606	0.071	**0.032**	**0.003**
PFOS						
Q1	Ref	Ref	Ref	Ref	Ref	Ref
Q2	-0.09 (-0.37, 0.20)	-0.41 (-1.16, 0.34)	-0.06 (-0.21, 0.09)	-0.21 (-0.54, 0.11)	-0.23 (-0.49, 0.02)	-0.04 (-0.37, 0.30)
Q3	-0.08 (-0.37, 0.20)	0.23 (-0.47, 0.93)	0.08 (-0.07, 0.23)	0.14 (-0.18, 0.46)	-0.08 (-0.34, 0.17)	0.09 (-0.24, 0.42)
Q4	0.03 (-0.25, 0.32)	0.42 (-0.30, 1.13)	-0.03 (-0.18, 0.12)	0.02 (-0.30, 0.33)	-0.10 (-0.35, 0.15)	**-0.38 (-0.71, -0.05)**
P-trend	0.829	0.069	0.792	0.427	0.715	0.061
PFNA						
Q1	Ref	Ref	Ref	Ref	Ref	Ref
Q2	0.21 (-0.07, 0.50)	0.11 (-0.61, 0.83)	-0.05 (-0.20, 0.10)	-0.09 (-0.41, 0.23)	-0.05 (-0.31, 0.20)	-0.21 (-0.55, 0.12)
Q3	0.28 (0.00, 0.56)	0.01 (-0.70, 0.73)	-0.09 (-0.24, 0.06)	-0.05 (-0.36, 0.27)	-0.23 (-0.48, 0.02)	**-0.38 (-0.71, -0.04)**
Q4	0.03 (-0.25, 0.31)	0.47 (-0.25, 1.18)	-0.02 (-0.17, 0.14)	-0.05 (-0.37, 0.27)	-0.13 (-0.39, 0.12)	**-0.36 (-0.69, -0.02)**
P-trend	0.724	0.248	0.710	0.819	0.161	**0.022**
PFDA						
Q1	Ref	Ref	Ref	Ref	Ref	Ref
Q2	0.14 (-0.14, 0.43)	-0.33 (-1.05, 0.38)	0.02 (-0.13, 0.18)	0.12 (-0.20, 0.44)	-0.08 (-0.34, 0.17)	0.02 (-0.32, 0.36)
Q3	0.18 (-0.10, 0.47)	-0.27 (-0.98, 0.44)	-0.01 (-0.17, 0.14)	0.05 (-0.27, 0.37)	-0.25 (-0.51, 0.00)	-0.07 (-0.41, 0.27)
Q4	0.12 (-0.16, 0.41)	0.31 (-0.40, 1.02)	0.00 (-0.15, 0.15)	0.23 (-0.09, 0.55)	-0.09 (-0.35, 0.16)	-0.33 (-0.66, 0.00)
P-trend	0.376	0.344	0.910	0.217	0.278	**0.046**
PFHxS						
Q1	Ref	Ref	Ref	Ref	Ref	Ref
Q2	0.10 (-0.18, 0.39)	0.52 (-0.23, 1.27)	0.07 (-0.08, 0.22)	0.30 (-0.02, 0.62)	0.17 (-0.09, 0.42)	0.14 (-0.20, 0.48)
Q3	0.09 (-0.21, 0.38)	0.40 (-0.38, 1.18)	-0.09 (-0.24, 0.07)	0.05 (-0.28, 0.37)	0.01 (-0.25, 0.28)	-0.06 (-0.41, 0.29)
Q4	0.01 (-0.28, 0.30)	0.57 (-0.17, 1.30)	0.04 (-0.11, 0.19)	0.29 (-0.04, 0.61)	0.13 (-0.13, 0.39)	-0.22 (-0.57, 0.12)
P-trend	0.965	0.216	0.887	0.250	0.579	0.118
PFHpS						
Q1	Ref	Ref	Ref	Ref	Ref	Ref
Q2	-0.02 (-0.31, 0.26)	**0.81 (0.10, 1.51)**	0.15 (0.00, 0.30)	0.17 (-0.16, 0.49)	-0.02 (-0.28, 0.23)	-0.19 (-0.53, 0.14)
Q3	-0.03 (-0.33, 0.26)	0.38 (-0.37, 1.12)	0.11 (-0.05, 0.26)	0.07 (-0.26, 0.39)	-0.12 (-0.39, 0.14)	-0.14 (-0.48, 0.20)
Q4	0.01 (-0.28, 0.29)	**0.73 (0.06, 1.39)**	0.12 (-0.03, 0.27)	0.00 (-0.32, 0.32)	-0.03 (-0.29, 0.23)	**-0.38 (-0.72, -0.04)**
P-trend	0.981	0.108	0.216	0.857	0.662	**0.048**
PFDoA						

Q1	Ref	Ref	Ref	Ref	Ref	Ref
Q2	0.17 (-0.11, 0.45)	-0.01 (-0.71, 0.69)	0.09 (-0.06, 0.24)	-0.02 (-0.34, 0.30)	0.18 (-0.08, 0.43)	0.09 (-0.25, 0.42)
Q3	**0.34 (0.06, 0.62)**	0.40 (-0.31, 1.11)	0.14 (-0.01, 0.29)	0.01 (-0.32, 0.33)	0.08 (-0.17, 0.34)	0.08 (-0.26, 0.42)
Q4	0.22 (-0.07, 0.51)	-0.17 (-0.84, 0.50)	0.13 (-0.02, 0.29)	0.07 (-0.26, 0.40)	0.08 (-0.18, 0.34)	0.26 (-0.09, 0.61)
P-trend	0.078	0.846	0.070	0.666	0.736	0.178
PFUdA						
Q1	Ref	Ref	Ref	Ref	Ref	Ref
Q2	0.11 (-0.18, 0.39)	0.39 (-0.31, 1.09)	-0.05 (-0.20, 0.10)	0.10 (-0.22, 0.42)	-0.17 (-0.43, 0.08)	0.16 (-0.18, 0.50)
Q3	0.19 (-0.10, 0.47)	0.14 (-0.59, 0.87)	0.04 (-0.11, 0.19)	0.06 (-0.26, 0.39)	-0.12 (-0.38, 0.13)	0.13 (-0.21, 0.47)
Q4	0.08 (-0.21, 0.37)	**0.75 (0.02, 1.48)**	-0.02 (-0.17, 0.13)	0.09 (-0.24, 0.41)	-0.20 (-0.46, 0.06)	-0.10 (-0.44, 0.24)
P-trend	0.489	0.101	0.872	0.678	0.184	0.559

* Adjusted for age, BMI, age at menarche, and education.

Bold characters indicate significance, $P < 0.05$.

Table S8. Linear associations of PFAS isomers (divided in quantiles) with Th17 and Treg cytokines concentrations (ln-transformed) (n=198)*.

PFAS isomers	Th17		Treg
	IL-17	IL-22	TGF-β
PFOA			
Q1	Ref	Ref	Ref
Q2	0.03 (-0.36, 0.41)	-0.18 (-0.72, 0.35)	0.14 (0.00, 0.28)
Q3	0.04 (-0.34, 0.42)	-0.15 (-0.68, 0.39)	**0.19 (0.05, 0.33)**
Q4	0.12 (-0.27, 0.51)	**-0.62 (-1.16, -0.09)**	**0.18 (0.04, 0.32)**
P-trend	0.558	**0.036**	**0.008**
PFOS			
Q1	Ref	Ref	Ref
Q2	-0.19 (-0.58, 0.20)	-0.27 (-0.82, 0.28)	0.01 (-0.13, 0.15)
Q3	0.02 (-0.36, 0.41)	0.08 (-0.47, 0.62)	0.09 (-0.05, 0.23)
Q4	-0.25 (-0.63, 0.14)	-0.23 (-0.76, 0.31)	0.01 (-0.13, 0.15)
P-trend	0.404	0.705	0.637
PFNA			
Q1	Ref	Ref	Ref
Q2	-0.14 (-0.53, 0.24)	0.14 (-0.41, 0.68)	**0.15 (0.01, 0.29)**
Q3	-0.18 (-0.57, 0.20)	-0.30 (-0.83, 0.23)	0.14 (0.00, 0.28)
Q4	-0.23 (-0.61, 0.16)	-0.05 (-0.58, 0.48)	0.12 (-0.01, 0.26)
P-trend	0.251	0.513	0.110
PFDA			
Q1	Ref	Ref	Ref
Q2	-0.19 (-0.58, 0.19)	-0.22 (-0.75, 0.32)	0.03 (-0.11, 0.17)
Q3	-0.11 (-0.50, 0.28)	-0.42 (-0.96, 0.12)	**0.15 (0.01, 0.29)**
Q4	-0.26 (-0.64, 0.12)	-0.04 (-0.57, 0.50)	0.05 (-0.09, 0.19)
P-trend	0.262	0.722	0.230
PFHxS			
Q1	Ref	Ref	Ref
Q2	0.35 (-0.03, 0.74)	0.31 (-0.23, 0.85)	0.10 (-0.04, 0.24)
Q3	0.24 (-0.15, 0.63)	-0.43 (-0.98, 0.11)	0.09 (-0.06, 0.23)
Q4	0.16 (-0.23, 0.55)	0.02 (-0.52, 0.56)	0.11 (-0.03, 0.26)
P-trend	0.567	0.477	0.163
PFHpS			
Q1	Ref	Ref	Ref
Q2	0.00 (-0.39, 0.39)	0.25 (-0.29, 0.78)	**0.19 (0.05, 0.33)**
Q3	0.04 (-0.36, 0.43)	0.42 (-0.14, 0.98)	0.07 (-0.07, 0.21)
Q4	-0.10 (-0.48, 0.29)	0.08 (-0.46, 0.62)	0.12 (-0.02, 0.26)
P-trend	0.675	0.687	0.291
PFDoA			
Q1	Ref	Ref	Ref
Q2	**-0.42 (-0.80, -0.03)**	-0.47 (-1.01, 0.07)	-0.10 (-0.24, 0.04)
Q3	-0.13 (-0.51, 0.25)	0.11 (-0.43, 0.65)	-0.04 (-0.18, 0.11)
Q4	-0.33 (-0.72, 0.06)	0.27 (-0.27, 0.82)	-0.06 (-0.20, 0.09)
P-trend	0.267	0.102	0.679
PFUdA			
Q1	Ref	Ref	Ref
Q2	0.05 (-0.33, 0.44)	0.31 (-0.23, 0.84)	-0.01 (-0.15, 0.13)
Q3	-0.28 (-0.66, 0.11)	-0.05 (-0.59, 0.50)	0.02 (-0.12, 0.16)

Q4	-0.33 (-0.72, 0.05)	-0.16 (-0.70, 0.37)	0.09 (-0.05, 0.23)
P-trend	**0.032**	0.337	0.199

[*] Adjusted for age, BMI, age at menarche, and education.

Bold characters indicate significance, $P < 0.05$.

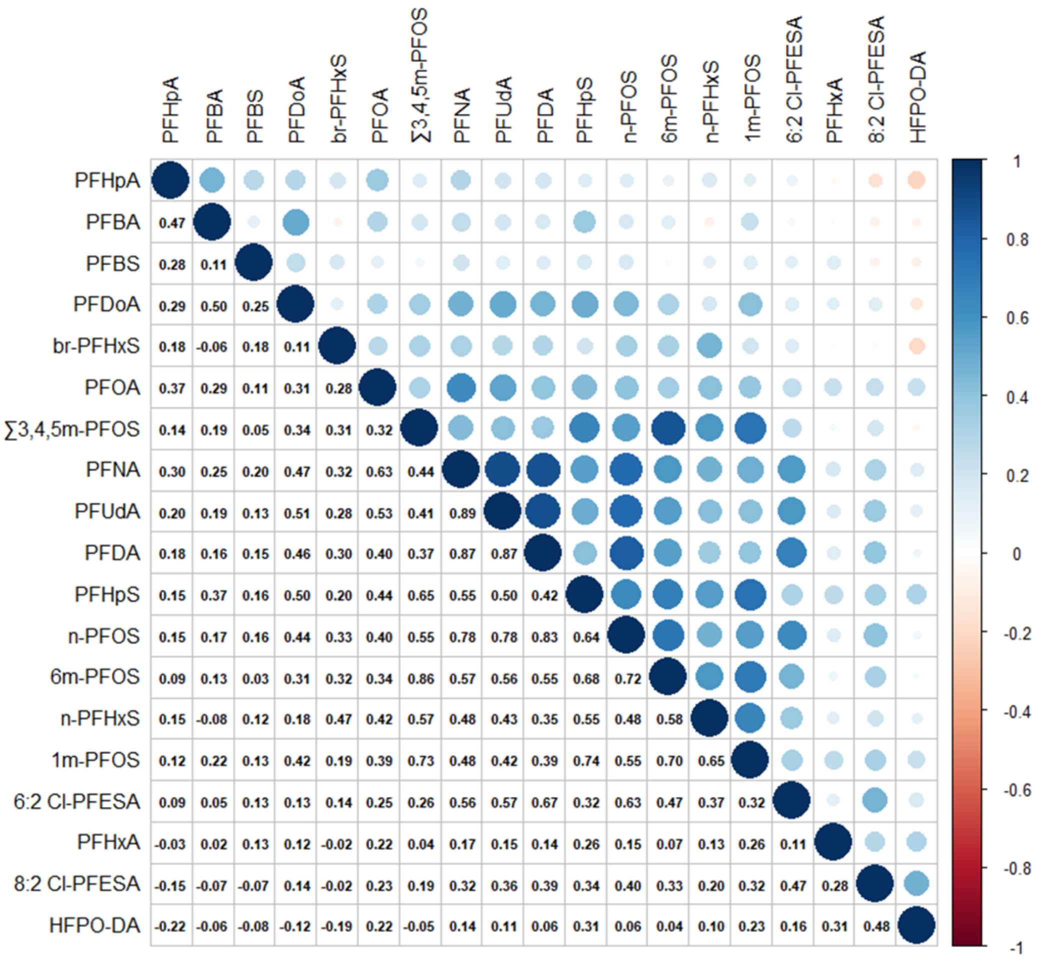

Figure S1. Correlations between plasma concentrations of PFAS.
